# Higher Dose Irradiation for Malignant Spinal Cord Compression: Long-Term Results of the RAMSES-01 Trial

**DOI:** 10.3390/curroncol33030149

**Published:** 2026-03-04

**Authors:** Dirk Rades, Darejan Lomidze, Natalia Jankarashvili, Fernando Lopez Campos, Arturo Navarro-Martin, Barbara Segedin, Blaz Groselj, Charlotte Kristiansen, Kristopher Dennis, Jon Cacicedo

**Affiliations:** 1 Department of Radiation Oncology, University of Lubeck, 23562 Lubeck, Germany; 2 Radiation Oncology Department, Tbilisi State Medical University and Ingorokva High Medical Technology University Clinic, Tbilisi 0177, Georgia; dlomidze@hotmail.com; 3 Department of Radiation Oncology, Acad. F. Todua Medical Center—Research Institute of Clinical Medicine, Tbilisi 0112, Georgia; natnataliaj@yahoo.com; 4 Department of Radiation Oncology, University Hospital Ramón y Cajal, 28034 Madrid, Spain; flcampos@salud.madrid.org; 5 Department of Radiation Oncology, Clínic Barcelona Villarroel 170, 08036 Barcelona, Spain; anavarroma@clinic.cat; 6 Department of Radiotherapy, Institute of Oncology Ljubljana and Faculty of Medicine, University of Ljubljana, 1000 Ljubljana, Slovenia; bsegedin@onko-i.si (B.S.); bgroselj@onko-i.si (B.G.); 7 Department of Oncology, Vejle Hospital, University Hospital of Southern Denmark, 7100 Vejle, Denmark; charlotte.kristiansen@rsyd.dk; 8 Division of Radiation Oncology, The Ottawa Hospital and the University of Ottawa, Ottawa, ON K1Y 4E9, Canada; krdennis@toh.ca; 9 Department of Radiation Oncology, Cruces University Hospital/Biobizkaia Health Research Institute, 48903 Barakaldo, Spain; jon.cacicedofernandezbobadilla@osakidetza.eus

**Keywords:** highly-conformal irradiation, local progression-free survival, long-term results, malignant spinal cord compression, propensity-score adjustment

## Abstract

Despite the increasing popularity of upfront decompressive surgery, there are still patients with malignant spinal cord compression (MSCC) and expected longer-term survival receiving irradiation alone. In these patients, local progression-free survival (LPFS) may be improved with doses beyond the commonly applied regimen of 10 × 3.0 Gy. A prospective phase 2 trial (RAMSES-01) investigated the benefit of two regimens, 15 × 2.633 and 18 × 2.333 Gy, compared with a 10 × 3.0 Gy (historical control). Patients in the phase 2 cohort had significantly better local progression-free survival (LPFS) after 1 year. Since recurrent MSCC-related motor weakness is a serious situation, it must be avoided as long as possible. In this respect, it is important to know whether the superiority of 15 × 2.633 and 18 × 2.333 Gy found in the RAMSES-01 trial still exists after 2 or 3 years. This led to the current study. In the phase 2 group, 2- and 3-year LPFS rates were 93.1% and 93.1%, respectively, and survival rates were 54.2% and 36.1%, respectively. According to propensity-adjusted Cox regression analyses, radiotherapy regimens in the phase 2 cohort resulted in significantly better LPFS at 2 (*p* = 0.017) and 3 (*p* = 0.013) years. In contrast, survival was not significantly different (*p* = 0.251 and *p* = 0.288, respectively). Radiation myelopathy and pathologic vertebral fractures were not observed in any group. Given the limitations of this study, irradiation 15 × 2.633 or 18 × 2.333 Gy may be an alternative option for patients with MSCC and longer expected survival treated with irradiation alone.

## Simple Summary

The RAMSES-01 trial compared two radiotherapy regimens, namely 15 × 2.633/18 × 2.333 Gy (phase 2 cohort) and 10 × 3.0 Gy (control group), in patients with malignant spinal cord compression (MSCC) not receiving upfront surgery and expected to be long-term survivors. Patients of the phase 2 cohort had significantly better local progression-free survival (LPFS) after 1 year. The question of whether this superiority would be present also after 2 or 3 years led to the present study. According to propensity-adjusted Cox regression analyses, LPFS was significantly better after 15 × 2.633 or 18 × 2.333 Gy at 2 years and 3 years when compared to 10 × 3.0 Gy. In both groups, radiation myelopathy or pathologic vertebral fractures were not reported. Given the limitations of this study, 15 × 2.633 or 18 × 2.333 Gy may be an alternative option for patients with MSCC and longer expected survival.

## 1. Introduction

Malignant spinal cord compression (MSCC) is a serious situation that may affect up to every tenth patient with solid cancers or hematological malignancies [1,2,3]. In the majority of patients presenting with MSCC, the expected survival time is only six months or less. However, in a considerable number of patients, the survival prognosis can be assumed to be much better, i.e., longer than 1 year. Standard treatment approaches include upfront decompressive surgery followed by irradiation and irradiation alone [1,2,3]. Upfront surgery is used in particular for patients with good performance status and an expected survival time of at least 3 months [4]. Irradiation alone is the preferred treatment for patients with a poor survival prognosis or a low performance status, as well as for patients with very radiosensitive tumors, paraplegia lasting for more than 48 h, or MSCC not restricted to a single area [4,5]. Since MSCC is regarded as an emergency situation, it is recommended that surgery be performed immediately [1,6]. If immediate surgery is not possible and the start of treatment is delayed, early irradiation is the preferred treatment [1,7]. Moreover, patients may not wish to undergo spinal surgery, which can be associated with complications [4], and opt for irradiation alone. Thus, many patients with MSCC are irradiated without preceding spinal surgery. Although stereotactic body radiation therapy (SBRT) has become quite popular for the treatment of spinal metastases in general, most patients with MSCC-associated neurologic symptoms receive conventional external-beam radiotherapy (EBRT) [8,9,10,11,12,13,14,15].

For these situations, a variety of EBRT programs exist [1,2,3,16,17]. The most common programs are 10 × 3.0 Gy in two weeks and 5 × 4.0 Gy in one week. Irradiation with one fraction of 8 or 10 Gy is also administered to patients with short expected survival times [18,19]. If patients are likely to live considerably longer than six months, they may be candidates for longer-lasting programs with higher total doses. Previous studies have suggested that irradiation with doses of 30 Gy or higher led to a better local progression-free survival (LPFS) than 5 × 4.0 Gy or 1 × 8.0 Gy, which is particularly important for long-term survivors [16,17,20]. This holds true for patients with a solid cancer and patients with myeloma [16,17]. Moreover, in a matched-pair analysis of 382 patients with MSCC and longer expected survival, total doses beyond 30 Gy (administered over three to four weeks) were associated with significantly better LPFS than 10 × 3.0 Gy [21].

To further investigate the value of increased doses to improve LPFS, the RAMSES-01 trial was performed, which compared 15 × 2.633 Gy or 18 × 2.333 Gy (phase 2 trial, 50 patients), delivered as highly conformal irradiation, with 10 × 3.0 Gy (historical control) [22]. Recruitment for the phase 2 trial started in August 2019 and was completed in November 2021. The follow-up period for the phase 2 trial ended 1-year after completion of irradiation for MSCC. For better comparability, the follow-up period was censored after one year in the control group. Patients of the phase 2 cohort had significantly better 1-year LPFS [22]. Since a recurrence of MSCC-related motor weakness is a serious situation for the patients, it should be avoided as long as possible. Thus, it is important to determine whether the regimens administered in the phase 2 group of the RAMSES-01 trial are still superior to 10 × 3.0 Gy two or even three years following irradiation. Therefore, this additional study was performed. Additional data (longer period of follow-up) were obtained for 32 of 50 patients (64.0%) in the phase 2 group and for 126 of 266 patients (47.4%) in the control group, which allowed the calculation of 2-year and 3-year LPFS-rates. This study aimed to contribute to decision making in patients with MSCC selected for irradiation alone, which is still a relevant clinical subgroup.

## 2. Materials and Methods

The multicenter phase 2 trial RAMSES-01 evaluated the value of irradiation alone with higher total doses (15 × 2.633 Gy or 18 × 2.333 Gy) in order to improve LPFS in patients with MSCC and longer expected survival times when compared to 10 × 3.0 Gy (historical control) [22]. The regimen in the original protocol of the RAMSES-01 trial was 18 × 2.333 Gy, representing an equivalent dose in 2 Gy fractions (EQD2) of 43.16 Gy_10_ for tumor cell kill (α/β ratio = 10 Gy). After the COVID-19 pandemic developed, it was decided to offer 15 × 2.633 Gy as an alternative regimen with a shorter overall treatment time. The corresponding EQD2 was 41.58 Gy_10_, i.e., only 3.7% lower than the EQD2 of 18 × 2.333 Gy. In the RAMSES-01 trial, highly conformal irradiation was performed using volumetric modulated arc therapy (41 patients) or intensity-modulated radiation therapy (nine patients). The planning target volume (PTV) was required to include the vertebrae affected by MSCC (plus a margin of 1 cm above and below) and to be covered by the 95–isodose. The maximum relative doses allowed to the spinal cord were 101.5% of the prescribed dose for 18 × 2.333 Gy and 101.2% for 15 × 2.633 Gy. Both doses represented an EQD2 of 46.6 Gy_2_ for myelopathy (α/β ratio = 2 Gy). The mean doses for the esophagus, heart and lung were required to be <34 Gy, <26 Gy and  ≤7 Gy, respectively.

To be included in the original RAMSES-01 trial, the patients were required to have motor weakness in one or both legs as a consequence of MSCC of the thoracic or lumbar spine. The diagnosis of MSCC had to be supported by spinal imaging (magnetic resonance imaging preferred). The maximum duration of motor weakness prior to the start of irradiation was 30 days. In order to select the most appropriate treatment for the patients, they were presented to a neurosurgeon to evaluate the necessity of upfront surgery. If surgery was required and performed, the corresponding patient was not eligible for the RAMSES-01 trial. Administration of corticosteroids was recommended during the course of radiotherapy, which was tapered down after its completion. More details of the patient characteristics and further aspects of irradiation of both groups have been previously reported [22].

The RAMSES-01 trial was approved by the responsible Ethics Committee (University of Lübeck, Germany; code 18-360) on the 19th of March 2019 and first posted on clinicaltrials.gov (NCT04043156) on the 2nd of August 2019. A total of 50 evaluable patients were included in the phase 2 trial and were compared to 266 patients in the historical control group who were irradiated for motor deficits of the lower extremities due to MSCC between 1998 and 2022 and had favorable survival prognoses. In the phase 2 cohort, 13 patients received 18 fractions of radiotherapy. Twelve of these patients were treated with 18 × 2.333 Gy, and one patient with 15 × 2.333 plus 3 × 2.0 Gy (EQD2 = 42.0 Gy_10_). Of the 37 patients who received 15 fractions, 33 patients were treated with 15 × 2.633 Gy, three patients with 12 × 2.633 plus 3 × 2.333 Gy (EQD2 = 40.5 Gy_10_), and one emergency patient with 1 × 3.0 Gy followed by 12 × 2.633 and 2 × 2.333 (EQD2 = 41.3 Gy_10_). Patients in both the phase 2 cohort and the control group had positive survival prognoses. The primary objective was the 1-year LPFS, defined as at least no further progression of motor symptoms during the course of irradiation and absence of a recurrence of MSCC-related motor symptoms afterwards. Secondary objectives were (overall) survival; radiation-related toxicities; and the effect of irradiation on motor and sensory symptoms, gait function, ambulatory status, pain score, and distress score.

The current additional study was approved by the Ethics Committee in Lübeck (code 2025-404) on the 3rd of September 2025. Its major goal was to compare the 50 evaluable patients in the prospective phase 2 cohort to the 266 patients in the control group with respect to 2-year and 3-year LPFS. Secondary goals included 2-year and 3-year survival rates and the occurrence of radiation myelopathy. During the one-year RAMSES-01 trial, follow-up visits were performed at the end of the radiotherapy course and at one month, three months, six months, nine months, and 12 months after completion of the radiation treatment. After this period, the patients did not undergo a structured follow-up program but received a clinical examination and spinal imaging only in case of new or progressive neurologic symptoms. The same applied to the patients in the control group throughout their entire period of follow-up. The diagnosis of radiation myelopathy was based on magnetic resonance imaging findings in patients presenting with new or progressive neurological symptoms. The other secondary goals of the original RAMSES-01 trial [22] were not evaluated in this additional study, since no change in comparison to the status after one year was expected.

To reduce the risk of selection bias due to differences between the two groups regarding baseline variables, a propensity score adjustment was performed, as done in the original RAMSES-01 trial [22]. The variables were considered for this adjustment, namely age (≤64 vs. ≥65 years), gender, interval from first diagnosis of malignancy until MSCC (≤15 vs. >15 months), organ metastases (no vs. yes), other osseous metastases (no vs. yes), primary tumor site (breast vs. prostate vs. lung vs. other vs. hematological malignancies), dynamic of the occurrence of motor weakness (fast = 1–7 day vs. intermediate dynamic = 8–14 days vs. slower = more than 14 days), gait function at the start of irradiation (unable to walk vs. able to walk), number of vertebrae affected by MSCC (1–2 vs. 3 or more), and the Eastern Cooperative Oncology Group (ECOG) performance score (better = 0–2 vs. worse = 3–4). Moreover, the LPFS and survival curves were censored at two years and at three years, respectively, for better comparability between the two groups. Corresponding figures are provided, showing the estimated 2-year and 3-year LPFS and survival rates after propensity score adjustment. After estimation of the propensity scores, a Cox regression model was applied to enable a non-linear relation between the propensity score and the outcome via an ‘one-spline’ approach [23]. Because of the low number of expected events, common approaches, including one-to-one matching, stratification, and inverse probability of treatment weighting, were considered not appropriate. To reduce the risk of potential biases, the modeling approach was pre-specified in the protocol of the RAMSES-01 trial. A restricted cubic spline transformation was applied that consisted of cubic functions between the knots and a linear function in the tails. Five knots were placed at equally spaced percentiles of the log odds. For the statistical analyses of this additional study, version 9.4 of the SAS software (SAS, Cary, NC, USA) was used.

## 3. Results

In the original RAMSES-01 trial, 15 patients in the phase 2 cohort died within 1 year after radiotherapy [22]. For 32 of the remaining 35 patients (91.4%), additional data were collected for the present study. In the control group, 62 patients died within the first post-radiotherapy year [22]. For 126 of the remaining 204 patients (61.8%), additional data were obtained.

**Figure 1:**
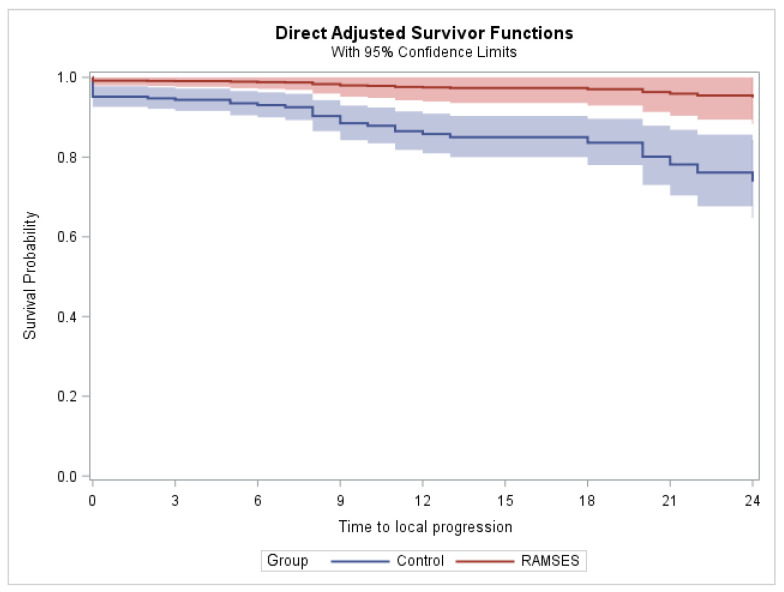
Estimated local progression-free survival (LPFS) after propensity score adjustment (censored at two years).

**Figure 2:**
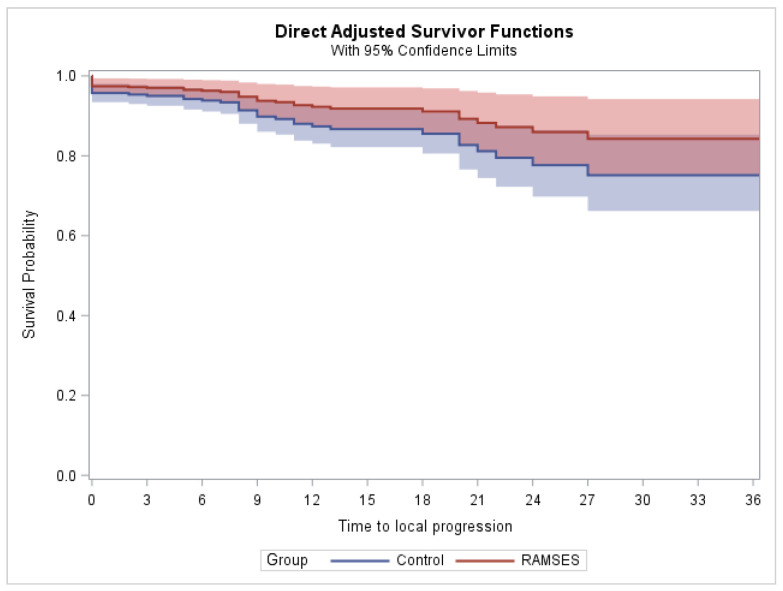
Estimated local progression-free survival (LPFS) after propensity score adjustment (censored at three years).

In the phase 2 cohort, two patients experienced local progression within two years after radiotherapy, and no additional case of local progression was found in the third post-radiotherapy year. In the control group, 35 patients were recorded to develop local progression within two years, plus one additional patient in the third year (total number = 36 patients). In the phase 2 group, the 2-year and 3-year LPFS rates (for the definition of LPFS, please refer to the Materials and Methods section) were each 93.1%. According to propensity-adjusted Cox regression analyses, the radiotherapy regimens used in the phase 2 cohort resulted in significantly better 2-year (*p* = 0.017) and 3-year (*p* = 0.013) LPFS rates. The Kaplan–Meier estimates for LPFS, censored at two and three years, are illustrated in Figure 1 and Figure 2, respectively. The results of the estimated group effects in the Cox regression model, both unadjusted and propensity score adjusted, are shown in Table 1.

**Figure 3:**
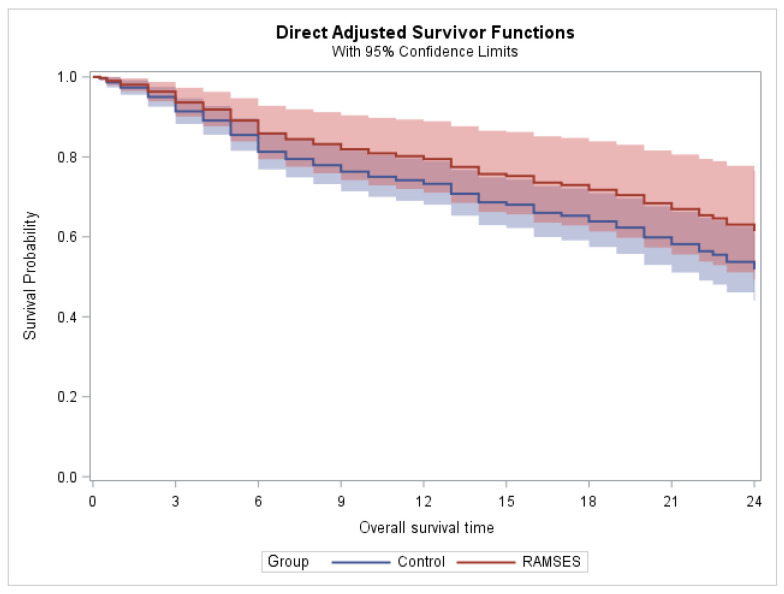
Estimated survival after propensity score adjustment (censored at two years).

**Figure 4:**
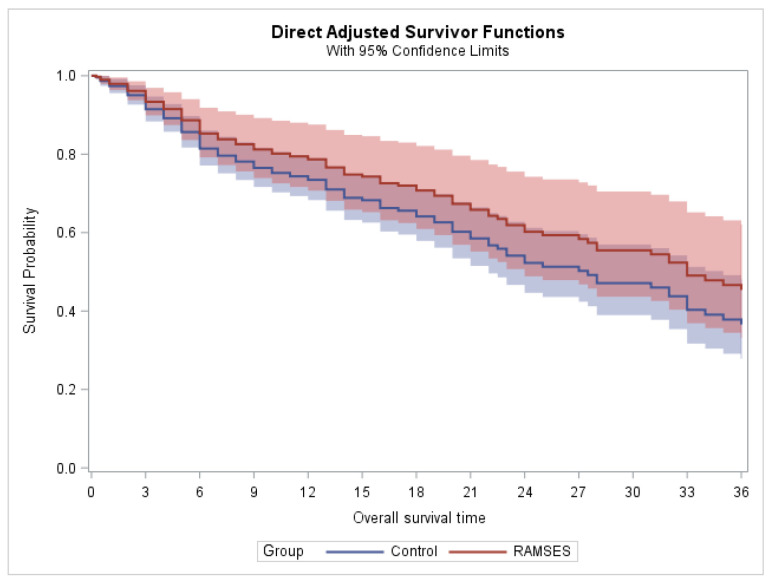
Estimated survival after propensity score adjustment (censored at three years).

In the phase 2 cohort, 22 patients died within two years after radiotherapy, and a total of 30 patients died within three years. In the control group, the corresponding numbers of deaths were 85 and 91, respectively. In patients belonging to the phase 2 group of the RAMSES-01 trial, the 2-year and 3-year survival rates were 54.2% and 36.1%, respectively. The corresponding Kaplan–Meier estimates for survival, censored at two and three years, are illustrated in Figure 3 and Figure 4, respectively. Propensity-adjusted Cox regression analyses did not reveal a significant difference between the phase 2 group and the control group with respect to 2-year and 3-year survival (*p* = 0.251 and *p* = 0.288, respectively). The results of the estimated group effects in the Cox regression model can be found in Table 2. Moreover, radiation myelopathy and pathologic vertebral fractures were not observed in any of the groups.

## 4. Discussion

MSCC is a serious situation that requires urgent treatment [24,25]. Most common treatment approaches are radiotherapy alone (often supplemented by the administration of corticosteroids) and spinal surgery followed by irradiation [1,2,3]. In 2005, a randomized trial suggested that in a specific group of patients irradiated for MSCC, treatment outcomes can be improved with the addition of neurosurgery [4]. In addition to other inclusion criteria, patients of that trial were required to be in good general condition and to have an estimated survival time of at least three months. The results have led to an increasing use of neurosurgical interventions, generally including decompression of the myelon and stabilization of the affected part of the spine [26,27,28,29,30,31,32]. Modern surgical approaches include the strategy of separation surgery [33,34,35]. Despite advances in spinal surgery, a large proportion of patients with MSCC are considered candidates for irradiation without surgery [1,2,3]. Most of these patients are either in a poor general condition, have a very limited life expectancy, or present with considerable comorbidity. However, a certain number of patients with a good performance status and other favorable factors at presentation are assigned to radiation therapy without upfront surgery. This group likely includes patients with very radiosensitive tumors or involvement of more than one area by MSCC [4,5]. Moreover, patients may refuse surgery, which bears a risk >10% of complications [4].

For patients with an expected survival time of one year or longer assigned to irradiation alone, the optimal dose fractionation is still under discussion. Previous studies found that multi-fraction programs with total doses of at least 30 Gy were superior to 5 × 4.0 Gy or 1 × 8.0 Gy in terms of LPFS [16,17,20]. Moreover, in a retrospective study of patients with a good survival prognosis, where 191 patients receiving 30 Gy (10 × 3.0 Gy) were thoroughly matched 1:1 to 191 patients irradiated with 37.5 Gy (15 × 2.5 Gy) or 40.0 Gy (20 × 2.0 Gy), LPFS was positively associated with the two higher-dose programs [21].

These findings were the rationale for the prospective RAMSES-01 trial, which demonstrated that irradiation with increased total doses resulted in better 1-year LPFS than 10 × 3.0 Gy [22]. Since the risk of experiencing a local (=in-field) recurrence of MSCC grows with increasing lifetime, it would be important to know whether the benefit of higher-dose irradiation still remains after two or three years. Therefore, this secondary analysis was performed with additional follow-up data in comparison with the original report of the RAMSES-01 trial [22]. According to its results, the regimens used in the phase 2 part of the RAMSES-01 trial, i.e., 15 × 2.633 Gy and 18 × 2.333 Gy, maintained their superiority regarding LPFS at two and at three years following irradiation. This may be explained by the biologically effective doses for tumor cell kill (alpha/beta ratio of 10.0 Gy) given as the equivalent dose in 2 Gy-fractions, which are 41.6 Gy for 15 × 2.633 Gy and 43.2 Gy for 18 × 2.333 Gy, respectively, and thus higher than for 10 × 3.0 Gy (32.50 Gy) [36]. Radiation myelopathy was not found in either of the two groups, which was not surprising, since the tolerance dose of the spinal cord was not exceeded by the dose-fractionation programs used for the phase 2 group and the control group [37]. The same held true for the occurrence of pathologic vertebral fractures. Unfortunately, the improvement in LPFS did not lead to better survival. However, since the cause of death is not known in the patients of the control group, this reason can only be a matter of speculation.

### Limitations

When interpreting these results, one needs to be aware of the limitations of this study.

The principal limitation is the non-randomized design with a retrospective historical control, creating unavoidable risks of confounding (e.g., changes in systemic therapy era, imaging availability, supportive care) and differential outcome ascertainment. In the phase 2 group, additional data were obtained for 32 patients of the 35 patients (91.4%) who were alive after 1 year in the original RAMSES-01 trial [22]. Since the trial was completed after a follow-up period of one year, these patients were not seen at the department participating in the RAMSES-01 trial at pre-defined intervals. However, they were seen irregularly by radiation oncologists or physicians of other disciplines at the participating hospitals or institutions. Therefore, at least for the 31 patients not developing a recurrence of MSCC later than one year following irradiation, the data can be considered valid. The only patient with a recurrence later than one year had no recurrence twelve months after treatment (date of the last follow-up of the RAMSES-01 trial) and experienced a recurrence only one month later. Therefore, this finding can be considered reliable, too. In the control group, where the follow-up period was extended beyond one year in 61.8% of the patients alive after 1 year in the original study, the situation is different. Since the data of this group were derived from an existing database, mainly including patients from retrospective studies, recurrences of MSCC may have been missed. This aspect may raise concern for informative censoring and differential detection of recurrences. However, more in-field recurrence would mean a worse LPFS. Thus, the long-term superiority of 15 × 2.633 Gy and 18 × 2.333 Gy demonstrated in the present study represents a more conservative scenario. One may speculate that the difference regarding LPFS between 15 × 2.633 Gy or 18 × 2.333 Gy and 10 × 3.0 Gy is even more pronounced.

Other potential limitations include the fact that the phase 2 part of the original RAMSES-01 trial was completed early after 50 evaluable patients (of 62 initially planned) [24]. Moreover, the fact that the formation of both dose groups was not performed in a randomized way and the retrospective nature of the data obtained for the control group need to be considered. Thus, hidden selection biases may have been introduced, although we aimed to reduce this risk by applying a propensity score-adjusted Cox regression model. Furthermore, it should be noted that the RAMSES-01 trial used conventional EBRT and, therefore, did not consider the option of SBRT, which is mainly used for reirradiation of spinal metastases but can also be of value for selected patients with single or a very limited number of spinal lesions causing MSCC [8,9,10,11,12,13,14,15,16,38,39,40,41,42,43,44]. Also, as survival improves, reirradiation becomes more frequent, even for malignant spinal cord compression in the form of a second course of EBRT, which can be performed safely [45]. The results of the present study may not apply to patients treated with SBRT or receiving irradiation after spinal surgery.

## 5. Conclusions

According to propensity-adjusted Cox regression analyses, irradiation with 15 × 2.633 Gy or 18 × 2.333 Gy resulted in significantly better LPFS after two and three years than 10 × 3.0 Gy, without showing an increased risk of myelopathy or pathologic vertebral fractures. Thus, the higher-dose programs may be an alternative option for patients with MSCC and longer expected survival times when assigned to irradiation alone. One should be aware of the limitations of this study when aiming to use 15 × 2.633 Gy or 18 × 2.333 Gy. Moreover, the results are not applicable to patients treated with SBRT or post-operative irradiation. A randomized trial is warranted to properly define the optimal dose-fractionation program for MSCC in patients with longer expected survival times.

## Figures and Tables

**Table 1:** Comparison of the phase 2 group cohort and the control group for local progression-free survival (LPFS) at two years and at three years—estimated group effects in a Cox regression model.

LPFS	Hazard Ratio	95% Confidence Interval	*p*-Value
At two years			
Unadjusted/crude	0.235	0.056–0.988	**0.048**
Propensity score adjusted	0.163	0.037–0.723	**0.017**
At three years			
Unadjusted/crude	0.212	0.050–0.893	**0.035**
Propensity score adjusted	0.150	0.034–0.667	**0.013**

Bold *p*-values are significant.

**Table 2:** Comparison of the phase 2 group cohort and the control group for survival at two years and at three years—estimated group effects in a Cox regression model.

Survival	Hazard Ratio	95% Confidence Interval	*p*-Value
At two years			
Unadjusted/crude	1.055	0.654–1.701	0.828
Propensity score adjusted	0.733	0.432–1.246	0.251
At three years			
Unadjusted/crude	1.071	0.694–1.653	0.755
Propensity score adjusted	0.774	0.482–1.242	0.288

## Data Availability

Data from the original RAMSES-01 trial can be found at clinicaltrials.gov (NCT04043156). Otherwise, the data used for the present article may not be shared because of the existing regulations of data protection.
